# Epithelial thymic tumours in paediatric age: a report from the TREP project

**DOI:** 10.1186/1750-1172-6-28

**Published:** 2011-05-21

**Authors:** Elena Carretto, Alessandro Inserra, Andrea Ferrari, Massimo Conte, Andrea Di Cataldo, Roberta Migliorati, Giovanni Cecchetto, Gianni Bisogno

**Affiliations:** 1 Division of Paediatric Surgery, Department of Paediatrics, Padova University Hospital, Padova, Italy; 2 Paediatric Surgery Department, IRCCS Ospedale Pediatrico Bambino Gesu`, Roma, Italy; 3 Paediatric Oncology Unit, Fondazione IRCCS Istituto Nazionale Tumori, Milano, Italy; 4 Department of Paediatric Haematology-Oncology, Giannina Gaslini Children's Hospital, Genova, Italy; 5 Department of Paediatric Haematology-Oncology, University of Catania, Catania, Italy; 6 Division of Paediatric Oncology, Pausilipon Children's Hospital, Napoli, Italy; 7 Haematology-Oncology Division, Department of Paediatrics, Padova University Hospital, Padova, Italy

## Abstract

**Background:**

Thymic epithelial tumours (thymoma and carcinoma) are exceptionally rare in children. We describe a national multicentre series with a view to illustrating their clinical behaviour and the results of treatment.

**Methods:**

From January 2000 all patients under 18 years of age diagnosed with "*rare paediatric tumours*" were centrally registered by the Italian centres participating in the TREP project (**T**umori **R**ari in **E**tà **P**ediatrica [Rare Tumours in Paediatric Age]). The clinical data of children with a thymic epithelial tumour registered as at December 2009 were analyzed for the purposes of the present study.

**Results:**

Our series comprised 4 patients with thymoma and 5 with carcinoma (4 males, 5 females; median age 12.4 years). The tumour masses were mainly large, exceeding 5 cm in largest diameter. Based on the Masaoka staging system, 3 patients were stage I, 1 was stage III, 1 was stage IVa and 4 were stage IVb.

All 3 patients with stage I thymoma underwent complete tumour resection at diagnosis and were alive 22, 35 and 93 months after surgery. One patient with a thymoma metastasizing to the kidneys died rapidly due to respiratory failure.

Thymic carcinomas were much more aggressive, infiltrating nearby organs (in 4 cases) and regional nodes (in 5), and spreading to the bone (in 3) and liver (in 1). All patients received multidrug chemotherapy (platinum derivatives + etoposide or other drugs) with evidence of tumour reduction in 3 cases. Two patients underwent partial tumour resection (after chemo-radiotherapy in one case) and 4 patients were given radiotherapy (45-54 Gy). All patients died of their disease.

**Conclusions:**

Children with thymomas completely resected at diagnosis have an excellent prognosis while thymic carcinomas behave aggressively and carry a poor prognosis despite multimodal treatment.

## Background

Primary thymic epithelial neoplasms, thymomas and thymic carcinomas, are uncommon tumours with an annual incidence of approximately 1-5 per million population [1]. Their aetiology is still largely unknown, but thymoma has been reported in association with multiple endocrine neoplasia syndrome type 1 (MEN1) and autoimmune disorders [2].

Thymoma is classified in two main types, depending on whether the neoplastic epithelial cells and their nuclei are uniformly bland (type A thymoma) or have a predominantly round or polygonal appearance (type B) [3]. Type B thymomas are further divided into three subtypes according to the extent of lymphocytic infiltrate and the degree of atypia of the neoplastic epithelial cells, i.e. B1 (richest in lymphocytes), B2, and B3 (richest in epithelial cells). Thymomas combining type A with B1-like or (rarely) B2-like features are designated type AB.

Thymic carcinomas are termed according to their differentiation (squamous cell, muco-epidermoid, etc.) [1].

In adults, the main histological subtypes in most published series are type B2 and AB thymomas (with 20-35% of all cases each) [4]. The percentage of thymic carcinomas is reportedly about 10-25% [5].

These tumours show variable clinical behaviour ranging from a tendency to be indolent and non-invasive to highly infiltrative tumours with metastatic spread to the pericardium and pleura and, occasionally, to the lung [6].

Thymic tumours are exceptionally rare in the paediatric age group, accounting for less than 1% of childhood mediastinal tumours [7]. As in adults, thymic tumours in children may be asymptomatic or present with compressive symptoms. They are classified and staged in the same way as adult tumours but there are very few reports on the treatment of thymic tumours in children.

With a view to promoting research on, and improving the clinical management of very rare paediatric cancers (childhood solid malignancies characterised by an annual incidence <2/million and not considered in other clinical trials) a nationwide cooperative initiative called the TREP project (Tumori Rari in Età Pediatrica [*Rare Tumours in Paediatric Age*]) was launched in Italy in 2000 [8,9]. Thymic tumours were included in this project and here we describe the clinical features, treatment and outcome of the TREP series patients with this diagnosis.

## Methods

All patients under 18 years of age with a diagnosis of "*rare paediatric tumours*" were centrally registered from 1 January 2000 onwards by all the Italian centres participating in the TREP project. The present study focuses on the clinical data recorded for 9 children with a histologically confirmed diagnosis of epithelial tumour of the thymus treated between January 2000 and December 2009. The series included 4 males and 5 females, with a median age of 12.4 years (range 4.8 - 16.3 years).

### TREP diagnostic and therapeutic recommendations

Guidelines were developed to help paediatric oncologists diagnose thymic tumours. After chest X-ray, computed tomography (CT) and/or magnetic resonance imaging (MRI) were recommended to assess local tumour extent. A bone marrow biopsy was part of the diagnostic work-up to rule out lymphatic neoplasms. Surgical guidelines suggested that primary excision should be attempted via a sternotomy if non-mutilating, and complete resection was considered feasible; if not, a biopsy was to be taken for diagnostic purposes. The criteria adopted for diagnosing thymoma or thymic carcinoma were as stated by the WHO classification. Tumours were staged according to the commonly-used Masaoka staging system [10] (Table 1). The distinction between tymoma and thymic carcinoma was based on the local pathology report. In addition 5 cases were reviewed by the TREP Pathology Panel and no discrepancies with the local diagnosis were noted.

**Table 1:** Thymoma staging according to Masaoka et al. [10]

Stage I	Totally encapsulated
Stage II	Capsular invasion and/or invasion into surrounding fat or pleura

Stage III	Invasion into organs (pericardium, lung, great vessels)

Stage IV-A	Pleural or pericardial implants

Stage IV-B	Haematogenous metastases

The paucity of data in the literature prevented us from establishing a strict protocol, but radiotherapy and chemotherapy were recommended in an attempt to reduce the tumour mass and make delayed surgery feasible. The guidelines stated that the most often used multidrug regimens included cisplatin, doxorubicin, vincristine and cyclophosphamide.

## Results

### Patients

The patients' demographic data are shown in Table 2. Eight children in our series were symptomatic, pain being the most frequent symptom (in 5 patients). Compression of the respiratory tract by the mediastinal tumour caused chronic coughing in 2 patients and dyspnoea in 2 (with airway compression and coma in 1). General symptoms (fatigue, fever, weight loss) were observed in 5 patients. One child had 2 paraneoplastic autoimmune syndromes (systemic lupus erythematosus and hypertrophic pulmonary osteoarthropathy). In one asymptomatic child, the tumour was diagnosed incidentally, after a radiological assessment for scoliosis.

**Table 2:** Clinical characteristics and treatment

N	Tumour type	Age (years)	Stage	Symptoms	Size (cm)	Local extent	Lymph nodes	Metastasis	Surgery	CT	RT (Gy)	Outcome/FU (m)
1	Thymoma B1	4.8	I	Cough	>5 e <10	no	no	no	Complete resection at diagnosis	No	No	1°CR/35.2

2	Thymoma B1	12.4	IVb	Dyspnoea, coma				Kidney, bilaterally	No	No	No	Died

3	Thymoma B1	11.4	I	Incidental (radiography for scoliosis)	4×2.5×2	no	no	no	Complete resection at diagnosis	No	No	1°CR/93.40

4	Thymoma AB	15	I	Chest pain, fever	16×8×14	no	no	no	Complete resection at diagnosis	No	No	1°CR/21.7

5	Lympho-epithelioma-like carcinoma	11.7	III	Chest pain, joint pain, fever, butterfly rash, digital clubbing	12×9×6	Lung, lymph node	Mediastinal	no	Incomplete resection at diagnosis	Ifo-VCR+ACT (1 cycle) CDDP+VP16 (2 cycles)	50	Died/12

6	Carcinoma with neuroendocrine differentiation	15.5	IVb	Shoulder pain	10×6×12	Sternum, pleural/pericardial effusion	Latero-cervical, mediastinal	Bone	Lymph node biopsy at diagnosis	CDDP+ VP16 (7 cycles)	Yes	Died/17

7	Poorly differentiated carcinoma	14.7	IVb	Fever, vomiting, weight loss, abdominal and chest pain	>5	Lung	Mediastinal	Bone	Lymph node biopsy at diagnosis	CDDP+5FU (1 cycle) CDDP+ADR+VCR+CPM (5 cycles)	45	Died/10

8	Poorly differentiated carcinoma	16.3	IVb	Cough, fever, fatigue, back pain	>5 e <10	no	Latero-cervical	Liver, bone	Lymph node biopsy at diagnosis	CDDP+CPM+ADR (4 cycle)	No	Died/7

9	Lympho-epithelioma-like carcinoma	12	IVa	Fatigue, weight loss, dyspnoea	10×10	Pleural nodes and effusion	Left hylar, mediastinal	no	Incomplete resection after chemotherapy	CDDP+5FU (4 cycles) CDDP (6 cycle)	54	Died/16

IFO = Ifosfamide, VCR = Vincristine, ACT = Actinomycin, CDDP = Cisplatin, VP16 = Etoposide, 5FU = Fluorouracil, CPM = Cyclophosphamide, ADR = Adriamycin, CT = chemotherapy, RT = radiotherapy, FU = follow-up (months), 1°CR = first complete remission

The tumour masses were generally large at diagnosis, exceeding 5 cm in largest diameter in 7 cases (and more than 10 cm in 4 of them).

The thymic carcinomas revealed a very aggressive behaviour with infiltration of adjacent organs (in 4 cases), regional lymph node invasion (in 5) and metastases to the bone (in 3) and liver (in 1). One child with thymoma had distant metastases to both kidneys.

According to the Masaoka staging system, 3 patients were classified as stage I, 1 as stage III, 1 as stage IVa and 4 as stage IVb.

### Treatment

A complete primary resection was performed at diagnosis in 3 patients with stage I thymoma, involving a sternotomy in 2 cases and a left thoracotomy in one.

In one child, the histopathological diagnosis of thymoma was established on autopsy after a rapid death due to respiratory failure resulting from compression of the respiratory tract by an unrecognized mediastinal tumour.

Among the 5 patients with carcinoma, a tumour resection was attempted in two (after chemo-radiotherapy in one), but left macroscopic residual disease; a biopsy of the enlarged regional lymph nodes was performed for diagnostic purposes in 3 cases. All received multidrug chemotherapy based on platinum derivatives in association with etoposide (2 cases) or other drugs. A tumour volume reduction was evident in three cases (see table: N 6, 8 and 9), ranging from 25% to 66%, but the response was short-lived (1 to 3 months).

Radiotherapy to the mediastinal region was administered in 4 patients, in doses ranging from 45 to 54 Gy.

### Outcome

The disease progressed in all 5 patients with carcinoma, who died from 7 to 17 months after its diagnosis. One child with thymoma also died soon after being admitted to hospital due to compression of the respiratory tract by the tumour mass. The 3 patients with stage I thymoma were alive with no evidence of disease 22, 35 and 93 months after complete tumour excision.

## Discussion

Our report confirms that thymic tumours are very uncommon in the paediatric age group.

In adults, the clinical behaviour of thymic tumours may vary from an indolent course to a very aggressive one. The WHO classification describes subtypes with a progressively worsening prognosis: thymoma types A, AB, and B1 have a relatively good outcome; B2 and B3 are more aggressive and have intermediate survival rates, while thymic carcinoma carries the worst prognosis [3].

For thymomas, the WHO histological classification and the Masaoka staging system are independent prognostic factors. Based on the Masaoka staging system, the 20-year survival rates are reportedly 89%, 91%, 49%, 0%, and 0% in patients with Stages I, II, III, IVa, and IVb disease, respectively [11].

Surgical resection is the mainstay of treatment for patients presenting with Masaoka Stage I or II disease; complete tumour resection is accompanied by complete thymectomy, the removal of all surrounding mediastinal fat and possibly also the pleura, to increase the chances of ensuring negative surgical margins [12].

Patients achieving a complete resection of a stage I tumour have a 5-year survival rate of 100% and a recurrence rate of 1% [13]; these patients are not considered candidates for adjuvant therapies.

Our limited experience suggests that thymomas in children have a similar behaviour with stage I thymoma having a favourable outcome. No further treatment was necessary in our cases after tumour removal. Conversely, the child with stage IV thymoma died of disease even before any therapy could be attempted.

Published experiences of paediatric thymoma are limited, but report similar results: In agreement with our results, Dhall [14] reported on 2 cases of thymoma (Masaoka stage I) treated with complete resection: both patients were still disease-free 3 years after surgery. Liang [15] reported on 2 cases of thymoma and provided a comprehensive review and analysis of paediatric cases reported in the past 30 years (32 cases in all): among 17 patients with stage I and II tumours, 16/17 patients (94%) were alive when their case was published (with a follow-up ranging from 3 months to 9 years), whereas only 3 of 9 patients (33%) with stage IV disease survived.

In adults, thymic carcinomas have a more aggressive clinical course than thymomas and they are associated with a poor prognosis. They are frequently not amenable to radical resection at diagnosis so multimodal therapies (including neo-adjuvant or adjuvant chemo-radiotherapy) are employed. Overall, the results obtained in adults with thymic carcinoma are unsatisfactory, with a reported 5-year survival rate of around 50% and a mean survival of 2.5 years [13,16 ,17]. Subgroup analysis has revealed a significant difference in survival rates between patients achieving total versus subtotal resections, and between totally resected and inoperable groups. The survival rate also differed significantly between patients receiving radio-chemotherapy and those receiving radiotherapy alone, and between the former and those given no adjuvant therapy [13].

Igawa [18] conducted a retrospective study on the efficacy and safety of combined CBDCA (carboplatin) + paclitaxel therapy in 11 previously-untreated patients with unresectable advanced thymic carcinomas: the overall median survival time was 22.7 months and the 1-year survival rate was 62%.

Studies describing children with thymic carcinoma are scarce. Yaris [19] reviewed the English literature and described 15 cases under 18 years of age, most of whom presented with an anterior mediastinal mass and suffered from chest pain, cough, fever, weight loss, and respiratory distress. Radiologically, these tumours were often associated with pleural effusions and/or the involvement of neighbouring structures such as the pleura and pericardium. Nine patients died (8 of them with metastatic disease) within 1.5 to 15 months of their diagnosis, 4 were alive (2 of them without disease) from 1 to 12 years after their diagnosis.

A recent report from the Polish Rare Tumour group [20] described 9 children with thymic carcinomas: 2 were classified as Masaoka stage II, 5 as stage III, and 2 as stage IV. Only 1 patient underwent complete tumour resection at diagnosis, six received multidrug chemotherapy and 4 had radiotherapy. The outcome was dismal, and only 2 children were long-term survivors.

The cases of thymic carcinoma in our series all had unfavourable features: they all presented with large masses and evidence of local or distant spread. Only a diagnostic biopsy was performed in 3 patients and an attempt at tumour resection in 2 left macroscopic residuals. Despite the administration of chemotherapy and radiotherapy, these tumours remained unresectable and all patients died.

Four patients in our series received radiotherapy, but it failed to reduce the tumour bulk. In the series published by the Polish group, 4 children were irradiated and 2 of them (with stage II and stage III disease) were still alive, though one of them unfortunately developed severe neurological sequelae due to radiation-induced spinal damage.

Although thymic tumours seem to be sensitive to chemotherapy, the most effective regimen remains to be established. Different drug combinations have been used, generally based on cisplatin, doxorubicin, cyclophosphamide and prednisone [21].

In our experience, we only found evidence of a short-lived tumour response in 3 children treated with cisplatin based chemotherapy. Similar results were obtained by the Polish group, with 4 out of 5 assessable children showing a tumour response after initial chemotherapy. Different regimens were administered, however, making it hard to say which is the most effective combination [20].

More effective drugs are therefore needed and targeted molecular therapies might pave the way to new therapeutic options in patients with thymic carcinoma in advanced stages. Strobel et al. [22] described the first case of a carcinoma with an activating KIT mutation and suggested that screening for activating KIT mutations may identify KIT-expressing carcinomas that could benefit from imatinib. The same authors [23] described 4 patients of metastatic thymic carcinoma refractory to conventional therapies who were treated with sunitinib, a multi-targeted tyrosine kinase inhibitor; 2 patients were still in partial remission after 14 and 18 months on sunitinib, one patient died 4 months after starting sunitinib, and the last patient was alive with stable primary disease and hepatic metastases, and with a partial remission of coeliac lymph node metastases.

## Conclusions

Thymic tumours are very rare in the paediatric age group. Like their adult counterparts, children with thymomas that are completely resected at diagnosis have an excellent prognosis. Thymic carcinomas behave very aggressively, however, and the prognosis is poor. The TREP project has demonstrated that cooperative studies are feasible even on exceptionally rare tumours and this approach should be transferred to a more international level in an effort to establish the best treatment for these very rare tumours.

## Competing interests

The authors declare that they have no competing interests.

## Authors' contributions

GB substantially contributed to the conception and design of the study and approved the final version of the manuscript.

EC and AI were involved in drafting the manuscript and critically revising its intellectual content.

GC contributed to data analysis and interpretation, and critically revised the work.

AF, MC, AD, RM made substantial contributions to data acquisition.

All authors have read and approved the final manuscript.
